# The influence of stationary synaptic activity on the PRC

**DOI:** 10.1186/1471-2202-12-S1-P264

**Published:** 2011-07-18

**Authors:** Guadalupe C Garcia, Gemma Huguet, John Rinzel

**Affiliations:** 1 School of Engineering and Science, Jacobs University Bremen, Bremen, Germany; 2 Center for Neural Science, New York University, New York, USA; 3 Centre de Recerca Matemàtica, Barcelona, Spain; 4 Courant Institute of Mathematical Sciences, New York University, New York, USA

## 

A useful measurable property of a neural oscillator is its Phase Response Curve (PRC). PRCs measure the phase-shift resulting from perturbing the oscillator with a brief stimulus at different times of the cycle. They have been extensively used to understand the synchronous activity patterns emerging from a network of weakly coupled oscillators.

PRCs have been classified into two types: type I (PRC is always positive) and type II (PRC has positive and negative regions) [1]. Theoretical results [2] have shown that the type of PRC combined with the temporal dynamics of the synapses yield different synchronization properties when two neurons are coupled together (neurons can synchronize in-phase, out of phase or in anti-phase).

PRCs are typically measured *in vitro*, considering only the intrinsic properties of the neuron. However, *in vivo* neurons constantly receive background synaptic inputs that play an important role sculpting the dynamics of neurons. Indeed experimental data showed that membrane excitability [3] can change in response to variations in background synaptic activity [4].

In this work we study the effects of the background synaptic activity on the shape of the Phase Response Curve, and its synchronization properties. To perform this study, we consider two neuron models: the Wang-Buzsáki model [5] and the Morris-Lecar model [6]. We explore the effect of a constant excitatory and inhibitory synaptic conductance input (that can be seen as an average of the background input) on the type of membrane excitability and PRC shape in the spiking regime.

We found that changes in the mean background conductances in a biologically plausible range [7] lead to changes in the type of PRC. As we increased the inhibitory conductance, for a constant value of the excitatory one, we observed a switch from type I to type II PRC. We correlated the shape of the PRC with the synchronization properties. We studied the effect of the temporal dynamics of synaptic activation on the synchronization properties of a coupled pair of neurons, as we switched them from type I to type II PRC. We characterized how solutions change with these parameters in a network motif of two reciprocally coupled neurons.
